# Study on natural breeding sites of sand flies (Diptera: Phlebotominae) in areas of *Leishmania* transmission in Colombia

**DOI:** 10.1186/s13071-015-0711-y

**Published:** 2015-02-22

**Authors:** Rafael José Vivero, Carolina Torres-Gutierrez, Eduar E Bejarano, Horacio Cadena Peña, Luis Gregorio Estrada, Fernando Florez, Edgar Ortega, Yamileth Aparicio, Carlos E Muskus

**Affiliations:** PECET (Program of Study and Control of Tropical Diseases), University of Antioquia, Medellín, Colombia; Grupo de Investigaciones Biomédicas, University of Sucre, Sincelejo, Colombia; Medical Entomology Unit, PECET University of Antioquia, Medellin, Colombia

**Keywords:** Immature, Breeding sites, Phlebotomine sand flies, Colombia

## Abstract

**Background:**

The location of the microhabitats where immature phlebotomine sand flies of the genus *Lutzomyia* develop is one of the least-known aspects of this group of medically important insects. For this reason strategies of source reduction approach for their control have not been possible in contrast to other insect vectors (such as mosquitoes), because their juvenile stages in terrestrial microhabitats is difficult to detect.

**Methods:**

Direct examination of soil samples, incubation of substrates and the use of emergence traps were the methods used to identify juvenile stages in 160 soil samples from urban and forest habitats within the foci of *Leishmania* transmission in Colombia. Immatures collected were identified subsequent from the rearing and emergence of adults using taxonomic keys or the analysis of the mitochondrial marker cytochrome oxidase I. Plant species associated with the natural breeding sites were identified and physicochemical properties of the soils were analyzed.

**Results:**

A total of 38 (23.7%) sampling sites were identified as breeding sites, 142 phlebotomine sand flies were identified, belonging to 13 species of the genus *Lutzomyia* and two of *Brumptomyia*. The greatest numbers of immature were found within the tabular roots (51 immature sand flies from eight positive sites) and bases of trees (35 immature sand flies from 11 sites). The characterization and presence of the tree species (mainly *Ceiba pentadra, Anacardium excelsum, Pseudosamanea guachapale*) and the physicochemical properties (relative humidity and carbon/nitrogen ratio) of the soils associated with these breeding sites are significant factors in explaining the diversity and abundance of phlebotomine sand flies.

**Conclusions:**

Immature phlebotomine sand flies of the genus *Lutzomyia* in Colombia can be found in a wide variety of breeding sites rich in organic matter, high relative humidity and are associated with a typical vegetation of each locality. These results provide new perspectives for the study of the ecology of the genus *Lutzomyia* in Colombia and the development of vector control strategies.

## Background

Protozoa of the *Leishmania* genus Ross 1903 (Kinetoplastida: Trypanosomatidae), are transmitted in the New World through the bite of dipteran insects belonging to the *Lutzomyia* genus (*Lu.*) França 1924, (Diptera: Psychodidae: Phlebotominae), indicating that their presence may represent an infection risk in the areas where they are found [1,2]. This group of zoonotic diseases occurs in at least 98 countries worldwide, principally in tropical and subtropical regions [1]. Sand flies are divided into three genera in America, with all known *Leishmania* vectors located in the genus *Lutzomyia* [2]. Some *Lutzomyia* species also transmit other pathogens of human and veterinary concern, such as the bacterium *Bartonella bacilliformis* Strong et al., 1907 and arboviruses (Phlebovirus and Vesiculovirus), responsible for bartonellosis, febril illness and vesicular stomatitis, respectively [3].

Approximately 500 species of phlebotomine sand flies have been recorded from the Americas, of which at least 164 are known to exist in Colombia, widely distributed in a variety of ecological environments [4-6]. In Colombia, thirteen species of *Lutzomyia* are associated or implicated with the transmission of different *Leishmania* species [7,8]. Cutaneous leishmaniasis is a health problem that affects people of all ages, often causing disfigurement that may have major psychological, socio-cultural and economic impact. Between 2005 and 2011, 63612 cases of leishmaniasis were recorded in Colombia, with an annual mean of 9087 cases, a significant increase being noted during the last three years [9].

With no vaccine available for humans, measures used by public health authorities to control leishmaniasis have focused on control of adult sand fly populations, for example by residual spraying with insecticides in and around human dwellings or the use of insecticide-impregnated bednets [10,11].

Systematic control of other insect vectors such as mosquitoes (vectors of malaria and dengue), is often aimed at the larval stages in aquatic breeding sites and adult stages. However these strategies have not been possible in the control of sand flies, because their development in terrestrial microhabitats is difficult to detect.

Although phlebotomine breeding sites have been identified by several workers [12-15], the microhabitats exploited by these insects remain little-known and hard to find. Several studies that attempt to produce mortality to larva of phlebotomines in the laboratory have included bacterial application (*Bacillus thuringiensis* and *B. sphaericus*) [16,17], fipronil-treated rodent baits used to reduce the populations of sand fly larvae [14], ground-up leaves of Neem or ivermectin [14], whereas Kumar et al. [18] tried to modify the properties of soil in breeding sites of *Phlebotomus* species by adding lime. All of these applications, could be significant, practical and cost-effective approaches to *Leishmania* vector control in situations in which breeding sites could be precisely located and characterized [15].

The difficulties in identifying the breeding sites of sand flies in rural areas where leishmaniasis transmission occurs have been attributed to the small size of the larvae and their sluggish movements [19], as well as to the great variety of sites that contain decomposing organic material in which the larvae can develop, making them difficult to perceive [12,20,21]. Other factors that impede the detection of sand fly larvae include the opportunistic behavior of the adults, which allows them to colonize diverse habitats, as well as the numerous interactions of phlebotomines with different tree species offering varied sources of food, protective refuges and physicochemical conditions favorable to larval development [21-24]. Given the lack of knowledge of the biology of immatures, detailed analysis of microhabitat characteristics that could regulate the reproduction, abundance and distribution of sand flies could be a useful precursor to studies of population dynamics and species richness, providing information that could be used in the design of vector control strategies [15,24,25].

This premise and the problems previously discussed have motivated new initiatives directed toward the study of immature stages of phlebotomines. Three recent studies in South America are particularly noteworthy: Alençar et al. [21] and Sangiorgi et al. [25], working in the Brazilian states of Amazonas and Bahia, respectively, found immature of *Lutzomyia* in the bases and roots of trees, while Parras et al. [26] collected specimens from the base of a bromeliad and from other substrates close to areas used by wild canines, in a study of visceral leishmaniasis in Chaco province, Argentina [26]. Recently, Souza et al. [27] found natural breeding sites of different species of sand flies on Marambaia Island (State of Rio de Janeiro) that participate in the transmission of leishmaniasis. The only Colombian record of immature stages and their breeding sites refers to *Lu. longipalpis* (Lutz & Neiva, 1912) [28].

The objective of the present study was to identify and characterize the natural breeding sites used by immature stages of *Lutzomyia* in rural and urban ecosystems of Colombia with previous records of the occurrence of leishmaniasis.

## Methods

### Study areas

**Figure 1:**
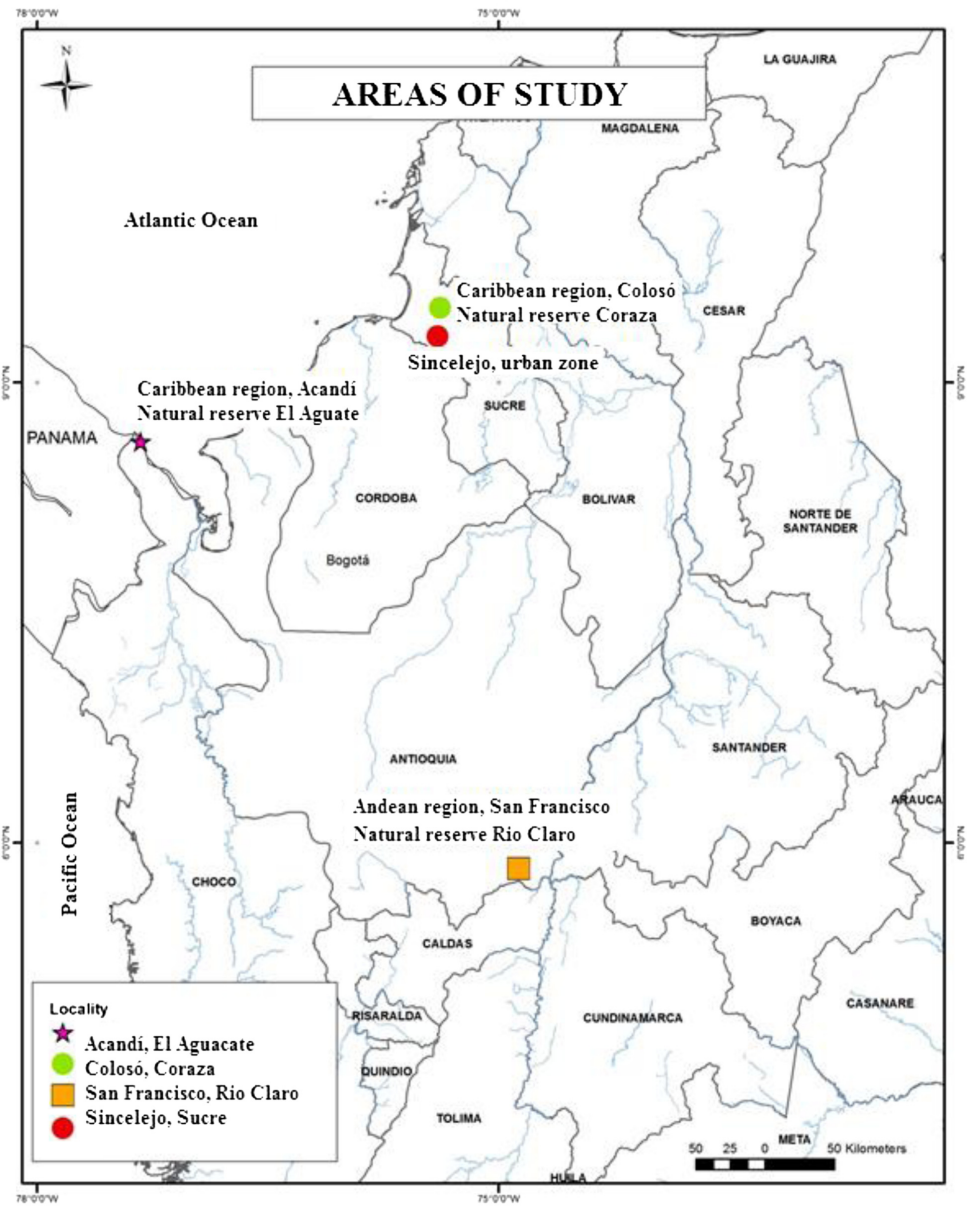
**Location of the four study areas selected for the search for natural breeding sites of Phlebotomine sand flies in Colombian foci of** ***Leishmania*** **transmission.**

Each locality was selected based on the incidence of leishmaniasis in Colombia during the last three years [9] and historical records of adult phlebotomines [4,29-32]. Two areas containing natural reserves were included and are classified as humid tropical forest ecosystems (**HTF**): Natural reserve Río Claro (5° 49′ 59. 37″N - 74° 52′ 00.62″W, 418 masl) located within the municipality of San Francisco (department of Antioquia, Andean region) and natural reserve El Aguacate (8° 36′ 53.85″N - 77° 19′ 39 15″W, 13 masl) located in the municipality of Acandí (department of Chocó, Caribbean region) (Figure 1).

To represent tropical dry forest ecosystems (**TDF**), we included the forest reserve of Serranía de Coraza in the Montes de María (Natural reserve Coraza) (09° 31′48.0″ N - 75° 21′ 4.3″W, 220 masl) situated in the municipality of Colosó and the urban zone of the city of Sincelejo (9° 17′ 42″N - 75° 23′ 46″W, 209 masl) (Figure 1), both sites in the department of Sucre, Caribbean region [32,33]. Entomological sampling efforts and the periods of time involved were different for each geographical locality. In Acandí (Chocó) sampling was carried out in October 2008 and September 2009; in San Francisco (Antioquia) during the months of May, June and August of 2008; in the city of Sincelejo in May, June, July, November, December of 2009; and in Colosó in May, June, July, August, September, November and December of 2009.

### Sampling in potential natural breeding sites and search for immature stages

The exploration and search for breeding sites of phlebotomine sand flies was based from published records or lists reported for insects of genus *Lutzomyia* in American tropical environments. Because of the wide range of habitats of these insect vectors, adult collections in diurnal resting places by active search with mouth aspirator were conducted, examining the rotation of genitalia in males and the physiological state (feeding status, gravid females) in individuals as search criteria for potential breeding sites (data not shown). It is likely that there are other types of microhabitats with conditions suitable for the development of immature sand flies in the areas explored in this study; this study only included a partial but significant representation of microhabitats, compared with previous publications [13,21,23]. Following the criteria previously outlined, 160 potential breeding sites of phlebotomine sand flies (**NBE =** *Number of natural breeding site examined*) were examined, 106 in dry tropical forest ecosystems (56 in Sincelejo and 50 in the natural reserve Coraza) and 54 in humid forest (36 in the nature reserve El Aguacate and 18 in Río Claro), corresponding to 11 different microhabitats (Table 1).

**Table 1:** **Potential breeding sites of phlebotomine sand flies examined and found to be positive**

**Ecosystem**	**Tropical dry forest**						**Tropical humid forest**								
**Region, department**	**Caribbean, Sucre**						**Andean, Antioquia**			**Caribbean, Chocó**					
**Locality**	**Sincelejo**			**Natural reserve Coraza**			**Natural reserve Río Claro**			**Natural reserve El Aguacate**			**Total**		
**Natural breeding site**	**NBE**	**NB** ^**+**^	**NIM**	**NBE**	**NB** ^**+**^	**NIM**	**NBE**	**NB** ^**+**^	**NIM**	**NBE**	**NB** ^**+**^	**NIM**	**NBE (%)**	**NB** ^**+**^ **(%)**	**NIM (%)**
1. Tabular roots	-	-	-	21	3	9	12	4	9	32	1	33	65 (40.6)	8 (21)	51 (35.9)
2. Base of tree	27	8	22	7	3	13	-	-	-	-	-	-	34 (21.2)	11 (29)	35 (24.6)
3. Tree hole	7	1	1	6	4	11	1	1	8	-	-	-	14 (8.7)	6 (15.7)	20 (14.1)
4. Bark of tree*	8	2	10	3	1	1	-	-	-	-	-	-	11 (6.8)	3 (7.8)	11 (7.7)
5. Leaf litter	4	3	7	3	2	4	3	-	-	-	-	-	10 (3.7)	5 (13.1)	11 (7.7)
6. Termite mound	2	1	6	-	-	-	-	-	-	1	-	-	3 (1.8)	1 (2.6)	6 (4.2)
7. Trunk of tree	2	1	2	4	2	3	-	-	-	-	-	-	6 (3.7)	3 (7.8)	5 (3.5)
8. Cave	-	-	-	2	1	3	2	-	-	-	-	-	4 (2.5)	1 (2.6)	3 (2.11)
9. Animal burrow	2	-	-	3	-	-	-	-	-	3	-	-	8 (5.0)	-	-
10. Axils of palms	2	-	-	-	-	-	-	-	-	-	-	-	2 (1.2)	-	-
11. Pig sties and chicken houses	2	-	-	1	-	-	-	-	-	-	-	-	3 (1.8)	-	-
**Total**	56	16	48	50	16	44	18	5	17	36	1	33	160 (100)	38 (100)	142 (100)
	106NBE/32NB^+^/92INM						54NBE/6NB^+^/50INM								

**NBE:** Number of potential natural breeding site examined, **NB** ^**+**^ **:** Positive natural breeding site with presence of immatures, **NIM:** Number of immatures found, *First record as natural breeding site of phlebotomine sand flies.

Each potential breeding site was sampled only once at a specific time, i.e., we did not perform an analysis of relative abundance of immatures (**NIM** = *Number of immatures*) in breeding sites (productivity) vs. seasonal alternation (future perspective of this study) because of the wide number of sites studied. Therefore, there is the possibility of differences in the search for immatures at each locality or the number of potential natural breeding sites examined. For this reason it is necessary to emphasize that the main purpose of this investigation was to detect and characterize natural breeding sites and inventory the species of immature phlebotomine sand flies present in a first attempt.

The methods used to isolate immature sand flies (larvae, pupae, and exuviae) and collect adults emerging from breeding sites were: (1) *direct examination* for the presence of immatures, which involved inspecting 500 g samples of soil from each microhabitat, dug to a depth of 15 cm in an area of 30 cm^2^. These samples were transported to the laboratory and preserved in 1 L polystyrene pots, maintained at a temperature between 26 - 29°C and a relative humidity (RH) between 80 - 90% until they could be inspected (2). *Incubation of substrates*, which involved preserving soil samples (about 500 grams) for 60 days in polystyrene pots (1000 cm^3^), under the conditions described above and monitoring them at 48 h intervals for the presence of newly emerged adult sand flies (3). *Emergence traps* were used only in the field in the Coraza reserve and in Sincelejo (10 traps were installed in each locality). The kind of emergence trap that was used is conical, covered with nylon and provided with one opening for the recovery of adults. The emergence traps were placed within the roots, bases of trees and surfaces of different substrates for 15 days, performing an inspection every three days in order to appreciate the emergence of adults. These methodologies have been used and described previously [12,13,21,25,26].

### Rearing and identification of immature stages

Larvae and pupae of phlebotomines recovered live by direct examination in the field were introduced individually into sterile 100 mL plastic containers that had been coated with a layer of plaster on the base. The diet provided to the larvae was a mixture of ground-up bovine faeces, fish food, liver powder and fruit of the chontaduro palm *Bactris gasipaes* [34]. These ingredients were combined with the original substrate collected in the field in a proportion of 1:1 and the plastic containers maintained under laboratory conditions. Any adults obtained from the rearing of immature specimens collected in the field or emerging from substrate samples, as well as those found in emergence traps placed on top of potential breeding sites in the field, were collected using mouth aspirators and preserved in isopropanol at −20°C before subsequent identification using taxonomic keys [35,36]. Immatures that died during the substrate incubation period in the laboratory were identified by means of gene amplification, sequencing and analysis of the mitochondrial marker cytochrome oxidase I (COI), using the DNA barcode for inventories of animal diversity [37,38] (Results not shown). Immatures that could not be identified were assigned to the genera *Lutzomyia* or *Brumptomyia* França & Parrot, 1921 based on the number of caudal setae on the terminal segments of IV instar larvae and pupae [19,35].

### Identification and characterization of tree species

Plant species associated with the natural breeding sites recorded for immatures of phlebotomine sand flies were identified by an expert, with additional information supplied by local people in each locality and literature on native species in each region. The following characteristics were noted for each tree species: circumference at breast height, type of bark (smooth, rugose or laminar); number, depth and length of tabular roots.

### Physico-chemical characterization of microhabitats

The temperature and relative humidity was determined at the moment of the collection for each microhabitat using a digital thermohygrometer fitted with a probe. For the physico-chemical characterization, 1 kg of soil was extracted from each positive breeding site to a depth of 20 cm. The samples were transported at 4°C and stored at −20°C for the determination of the following parameters: percentage of organic carbon using the Kjeldahl technique; water retention capacity; carbon/nitrogen (C/N) ratio; soil pH, conductivity and density.

### Statistical analysis

Phlebotomine sand flies present in the four localities and the two ecosystems, were correlated with tree species and their distinct morphologies, illustrating the associations for each habitat that exhibited a typical vegetation. The relationship with the morphological and physical characteristics of the plant species was evaluated and compared with the richness and relative abundance of phlebotomine sand fly species identified, by means of multiple discriminant correspondence analysis using the XLSTAT 3.04 program (http://www.xlstat.com/). Finally, data derived from the characterization of physicochemical parameters of the breeding sites in dry and humid forest ecosystems were initially analyzed with the Mann–Whitney test, using the SPSS 17.00 program (http://www-1.ibm.com/software/co/analytics/spss/products/statistics/). The measurements of physical and chemical parameters can be ordered on an ordinal scale, so this test is suitable for data that are not normally distributed and are acceptable for independent samples when considering each location as a particular habitat type. Additionally, the physical and chemical parameters compared to the richness and relative abundance of species was evaluated by multiple discriminative correspondence analysis, using the XLSTAT 3.04 program, to confirm or reject tendencies of association or grouping of phlebotomine species with these parameters. The multiple correspondence methods used in this study are object-categorization algorithms that use discriminative methods combined with global and/or local and scale variations. Additionally, recent work has shown the suitability of discriminative methods for recognition of large numbers of categories.

## Results

### Natural breeding sites

**Figure 2:**
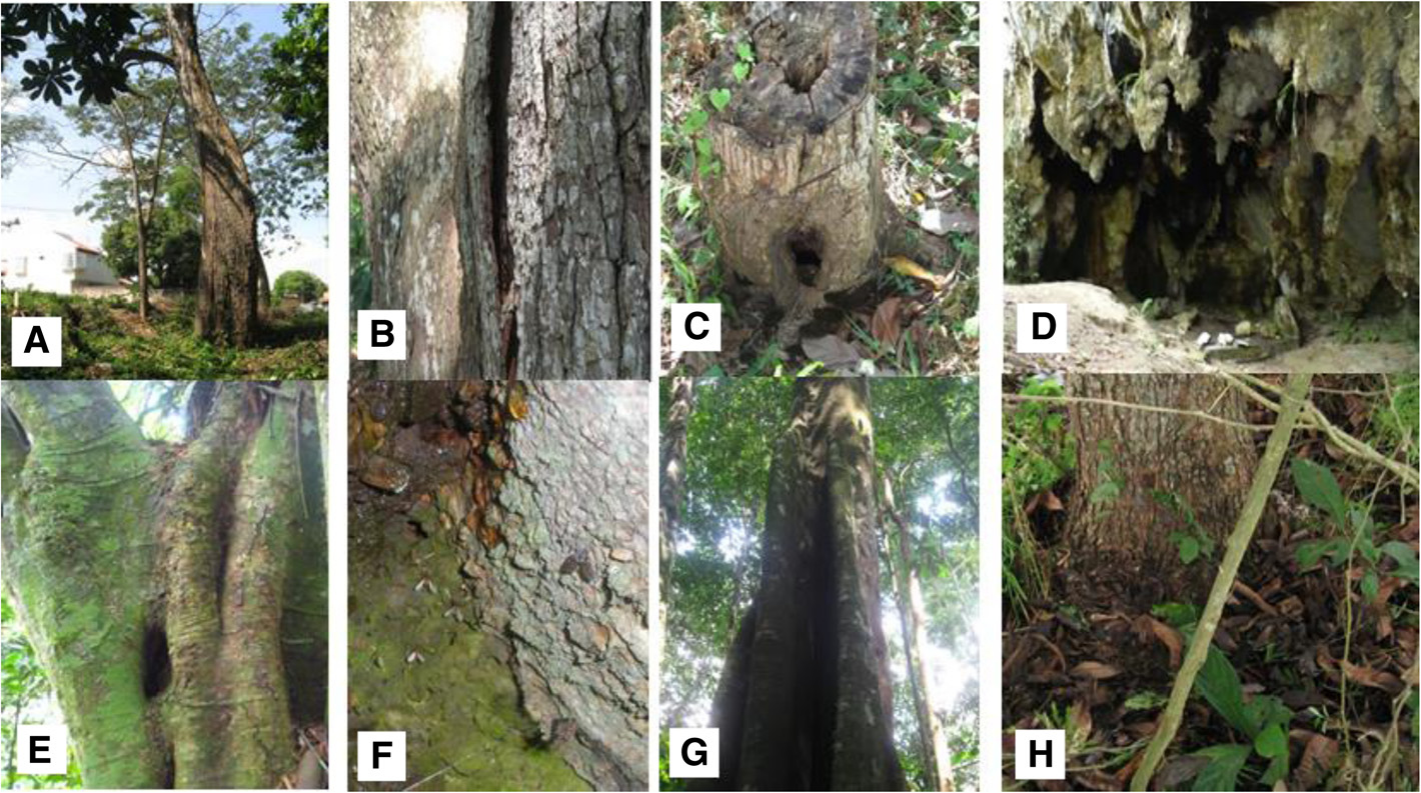
**Positive breeding sites of phlebotomine sand flies in four areas of** ***Leishmania*** **transmission in Colombia. A**. Base of tree, **B**. Bark of tree, **C**. Trunk of tree, **D**. Cave, **E**. Hollow of tree, **F**. Termite mound with resting phlebotomine sand flies, **G**. Area between tabular roots, **H**. Leaf litter.

A total of 38 (23.7%) sampling sites were identified as breeding sites, 32 of them in dry forest and the remaining six in humid forest ecosystems. The Sincelejo samples yielded the greatest number of insects (*NIM* = 48), followed by the Coraza, El Aguacate and Río Claro reserves (*NIM* = 44, *NIM* = 33 and *NIM* = 17, respectively; see Table 1). Direct examination allowed recovery of 104 immatures and eight exuviae of Phlebotomine sand flies (78.9% of the total collection), whereas 26 adults (18.3%) were obtained by incubation of soil samples in the laboratory and four (2.8%) were collected from emergence traps in the field (all of the latter in the Coraza reserve). Eight different microhabitat types yielded immature and/or emerged sand flies (Table 1, Figure 2), indicating high productivity in tabular roots (51 immature sand flies from eight positive sites), tree bases (35 immature sand flies from 11 sites), and tree holes (20 immature sand flies from six sites) (Figure 2). The leaf litter, trunk tree and a tree hole in Rio Claro (specifically) also have high produtivity, because the number of sites analyzed is lower, but detection results as positive microhabitat are important.

In dry forest ecosystems the tree bases (Figure 2) were the predominant natural breeding sites, particularly in Sincelejo (22 immature sand flies from eight positive sites). Only six breeding sites were detected in the two rural ecosystems of humid tropical forest, with 33 immatures collected from a single site in El Aguacate and 17 from one tree hole and four tabular roots (Figure 2) in Río Claro (Table 1).

### Species of phlebotomines found in natural breeding sites and tree species

Direct examination produced 93 larvae, 11 pupae and eight exuviae, of which 30 immatures (25 larvae; five pupae) completed their development to adult in the laboratory. The 30 immatures were identified together with the 30 adults obtained by incubation (n = 26) and the emergence traps (n = 4) using taxonomic keys [35,36]. In addition, 18 immatures found dead during examination of samples in the field were identified using the mitochondrial marker *COI* (Data not shown). Contrasting sequences of unknown identity of immatures and adult with predetermined morphological identity were found values of genetic distances (K2P) between 0,002 and 0,031. The supports bootstrapp in the dendrogram Neighbor Joining (>96%) are consistent for most MOTUS established. The COI gene is validated as a taxonomic marker for the definition and delimitation of species of the genus *Lutzomyia* (Data not shown).

Taxonomic tools were used to identify 78 phlebotomines (54.9%), corresponding to 13 species of the genus *Lutzomyia* and two species of the genus *Brumptomyia* (Table 2). The most abundant species were *Lu. atroclavata* (Knab 1913) (14 specimens; 9.8% of the total; in Sincelejo) and *Lu. migonei* (França 1920) (13 specimens; 9.1%; in the Coraza reserve dry forest). In the humid forest ecosystems of Río Claro and El Aguacate, most of the immatures collected were *Brumptomyia* species, with a few individuals of *Lutzomyia* (Table 2).

**Table 2:** **Species and relative abundance of immature phlebotomine sand flies found in natural breeding sites**

**Ecosystem**	**Tropical dry forest**							**Tropical humid forest**			
**Region, department**	**Caribbean, Sucre**							**Andean, Antioquia**		**Caribbean, Chocó,**	
**Locality**	**Sincelejo, urban area**			**Natural reserve Coraza**				**Natural reserve Río Claro**		**Natural reserve El Aguacate**	
**Phlebotomine sand flies**	**Base tree**	**Bark of tree**	**Others**	**Based of tree**	**Hollow of tree**	**Tabular roots**	**Others**	**Hollow of tree**	**Tabular roots**	**Tabular roots**	**Total (%)**
1. *Lu. atroclavata*	9	4	1	-	-	-	-	-	-	-	14 (9.8)
2. Lu. rangeliana*^+^	1	2	1	-	-	-	-	-	-	-	4 (2.9)
3. Lu. c. cayennensis*^+^	4	-	2	-	1	-	-	-	-	-	7 (4.9)
4. *Lu. micropyga*	2	-	1	-	-	-	1	-	2	2	8 (5.6)
5. *Lu. dubitans* ^+^	-	-	1	-	1	1	3	-	-	-	6 (4.2)
6. Lu. evansi*^+^	1	-	2	-	-	-	2	-	-	-	5 (3.5)
7. Lu. migonei*^±+^	-	-	-	6	-	6	1	-	-	-	13 (9.15)
8. *Lu. serrana*	-	-	-	3	1	-	1	-	-	-	5 (3.5)
9. *Lu. gorbitzi* ^±+^	-	-	-	-	1	-		-	-	-	1 (0.7)
10. Lu. ovallesi*	-	-	-	-	-	-	2	-	-	-	2 (1.4)
11. Lu. shannoni*^+^	-	-	-	-	2	-	-	-	-	-	2 (1.4)
12. Lu. trinidadensis*	-	-	-	-	-	-	-	-	2	-	2 (1.4)
13. *Lu. pilosa* ^+^	-	-	-	-	-	-	-	-	2	-	2 (1.4)
14. *Br. hamata*	-	-	-	-	-	-	-	2	-	4	6 (4.2)
15. *Br. mesai* ^+^	-	-	-	-	-	-	-	-	-	1	1 (0.7)
16. *Brumptomyia* sp.	-	-	-	-	-	-	-	-	-	26	26 (18.3)
17. *Lutzomyia* sp.	5	4	8	4	5	2	1	6	3	-	38 (26.8)
**Total**	22	10	16	13	11	9	11	8	9	33	142 (100)

**Others:** Trunk of tree, Leaf **l**itter, Termite mound, Cave, *Species associated with or incriminated in the transmission of *Leishmania*, ^+^First record of natural breeding sites for species of phlebotomine, ^±^New record of phlebotomine sand flies species for this department.

Species such as *Lu. cayennensis cayennensis* (Floch & Abonnenc, 1941), *Lu. serrana* (Damanesco & Arouck, 1949), *Lu. atroclavata*, *Lu. shannoni* (Dyar, 1929), *Lu. gorbitzi* (Blancas, 1959) and *Lu. migonei*, were collected in wide numbers of microhabitats associated with plant structures such as bark, holes and trunks of trees (Table 2). By contrast, *Brumptomyia* species (*B. hamata* Fairchild & Hertig, 1947 and *B. mesai* Sherlock, 1962) and some of genus *Lutzomyia* (*Lu. trinidadensis* (Newstead, 1922), *Lu. pilosa* (Damasceno & Causey, 1944) and *Lu. micropyga* (Mangabeira, 1942)) were mostly found in tabular roots of trees (Table 2). Some species of the genus *Lutzomyia* (n = 7) are medically important, among these are *Lu. migonei*, *Lu. serrana*, *Lu. ovallesi* (Ortiz, 1952), *Lu. rangeliana* (Ortiz, 1952), *Lu. c. cayennensis*, *Lu. shannoni*, and *Lu trinidadensis* (anthropophilic and zoophilic sand flies), which are suspected to be vectors of different species of *Leishmania* in several regions of their respective geographic ranges. *Lu. evansi* (Nunez-Tovar, 1924) is the proven vector of *Le. infantum* in northern Colombia [39-41].

### Tree species associated with breeding sites

A total of 26 species of trees were identified and associated with sand fly breeding sites (Table 3). In the tropical dry forest ecosystem 22 species of trees were identified (Sincelejo *C-SL* = 11; Coraza reserve C-CR = 11). Only six were found in the rural humid forest habitats, with five in Río Claro and the other in El Aguacate (Table 3). The species *Ceiba pentadra* Gaertn, 1971, which was recorded from all three wild ecosystems surveyed, yielded the greatest number of immature sand flies (*n =* 46), of which 31 were *Brumptomyia* species and 15 belonged to the genus *Lutzomyia* (Table 3). Two other tree species (*Anacardium excelsum* Bertero & Balb ex Kunth, 1912 and *Pseudosamanea guachapale* Kunth 2002), identified in Sincelejo, proved important in the productivity of immatures (Table 3).

**Table 3:** **Species of tree where immature Phlebotomine sand flies were found**

	**Region-locality**				**Phlebotominae sand flies**		
**Tree species**	**C-SL**	**C-CR**	**C-AG**	**A-RC**	**N** ***Lutzomyia*** **(%)**	**N** ***Brumptomyia*** **(%)**	**Total (%)**
1. *Pithecellobium dulce -* **FB**	X	-	-	-	2 (1.4)	-	2 (1.4)
2. *Cordia dentata -* **BR**	X	-	-	-	2 (1.4)	-	2 (1.4)
3. *Samanea saman -* **FB**	X	-	-	-	5 (3.5)	-	5 (3.5)
4. *Anacardium excelsum* - **AN**	X	-	-	-	13 (9.1)	-	13 (9.1)
5. *Mangifera indica* - **AC**	X	-	-	-	5 (3.5)	-	5 (3.5)
6. *Platymiscium pinnatum* - **FB**	X	-	-	-	2 (1.4)	-	2 (1.4)
7. *Pseudobombax septenatum* - **BB**	X	-	-	-	1 (0.7)	-	1 (0.7)
8. *Crescentia cujete* - **BG**	X	-	-	-	4 (2.8)	-	4 (2.8)
9. *Cordia bicolor* - **BR**	X	-	-	-	1 (0.7)	-	1 (0.7)
10. *Pseudosamanea guachapele -* **FB**	X	-	-	-	10 (7.0)	-	10 (7.0)
11. *Cedrela odorata -* **ML**	X	-	-	-	1 (0.7)	-	1 (0.7)
12*. Nectandra membranaceae* - **LR**	-	X	-	-	2 (1.4)	-	2 (1.4)
13. *Myroxylon balsamum -* **FB**	-	X	-	-	2 (1.4)	-	2 (1.4)
14. *Trichilia acuminata -* **ML**	-	X	-	-	2 (1.4)	-	2 (1.4)
15. *Aspidosperma megalocarpon* - **AP**	-	X	-	-	4 (2.8)	-	4 (2.8)
16. *Brownea ariza* - **FB**	-	X	-	-	2 (1.4)	-	2 (1.4)
17. *Soroceae sprucei -* **MR**	-	X	-	-	1 (0.7)	-	1 (0.7)
18. *Ceiba pentandra -* **BB**	-	X	X	X	15 (10.5)	31 (21.8)	46 (32.3)
19. *Basiloxylum excelsum -* **ST**	-	X	-	-	1 (0.7)	-	1 (0.7)
20. *Dilodendron sp. -* **SP**	-	X	-	-	1 (0.7)	-	1 (0.7)
21. *Heisteria acuminata* - **OL**	-	X	-	-	2 (1.4)	-	2 (1.4)
22. *Albizia nipoides -* **MM**	-	X	-	-	1 (0.7)	-	1 (0.7)
23. *Protium* sp. - **BS**	-	-	-	X	2 (1.4)	-	2 (1.4)
24. *Schizolobium parahyba* - **FB**	-	-	-	X	2 (1.4)	-	2 (1.4)
25. *Vochysia ferruginea -* **VC**	-	-	-	X	5 (3.5)	2 (1.4)	7 (4.9)
26. *Dialium guianense -* **FB**	-	-	-	X	2 (1.4)	-	2 (1.4)
27. ND	-	-	-	-	19 (13.3)	-	19 (13.3)
**Total (%)**	11	11	1	5	109 (76.7)	33 (23.2)	142 (100)

**C-SL:** Caribbean region, Sincelejo, **C-CR:** Caribbean region, Nature reserve Coraza, **C-AG** Caribbean region, Nature reserve El Aguacate, **A-RC:** Andean Region, Nature reserve Rio Claro, **FB:** Fabaceae, **BR:** Boraginaceae, **AN:** Anacardiacerae, **AC:** Anacardiaceae, **BB:** Bombacaceae, **BG:** Bignoniaceae, **ML:** Meliaceae; **LR:** Lauraceae, **AP:** Apocinacae, **MR:** Moraceae, **ST:** Sterculiaceae, **SP:** Sapindaceae, **OL:** Olacaceae, **MM:** Mimosaceae, **BS:** Burseraceae, **VC:** Vochysiaceae. **ND:** Plant species indeterminate.

**Figure 3:**
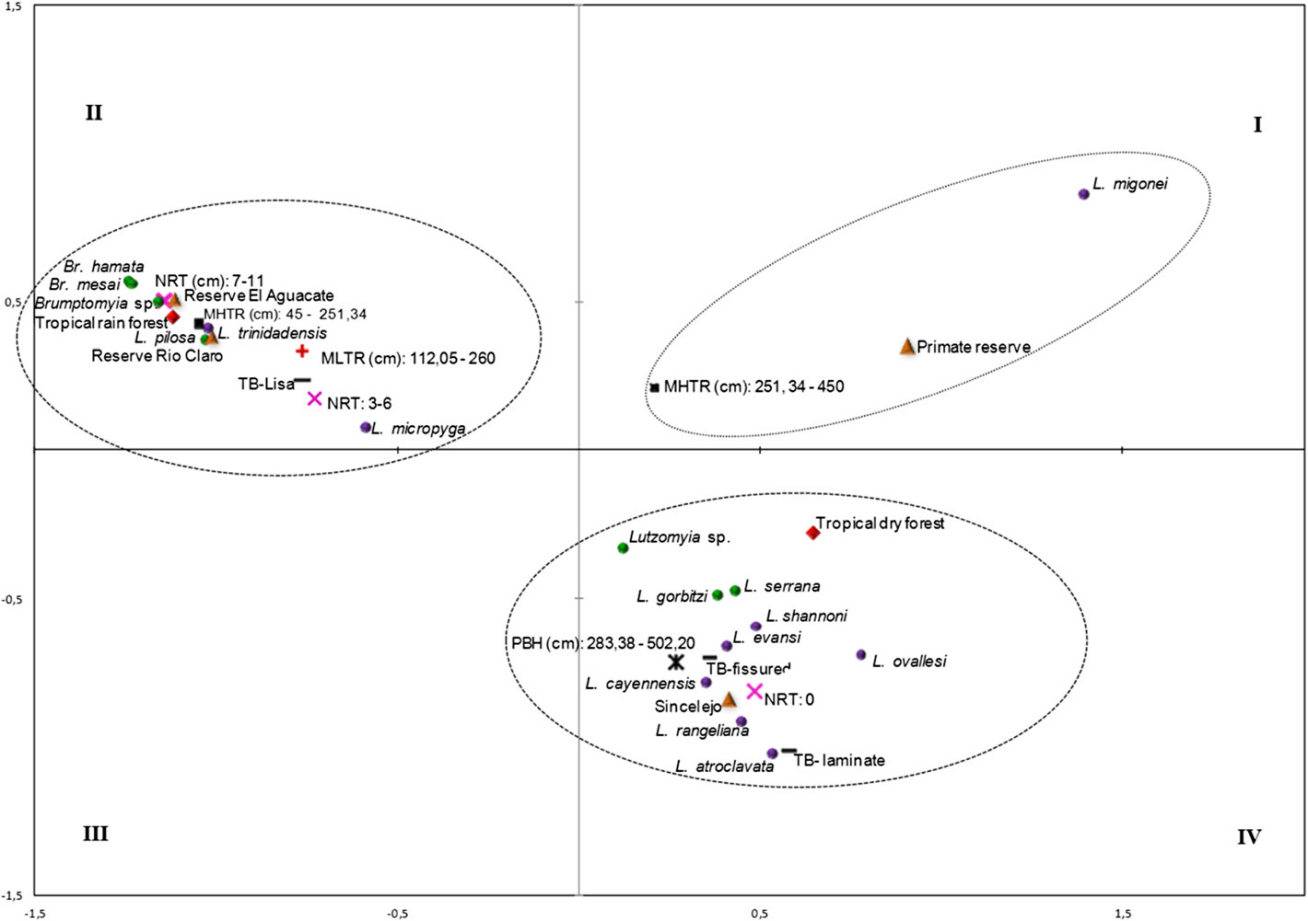
**Multiple correspondence analysis (foreground factorial axes F1 and F2, 75.31% of variance), which relates quantitative and qualitative morphological variables of tree species associated with natural breeding places of Phlebotomine sand flies.** Triangle diamond Locality, green circle Species of the subfamily Phlebotominae that are not vectors of *Leishmania*, purple circle Species of the subfamily Phlebotominae associated with or incriminated in the transmission of *Leishmania*, red diamond Ecosystem, black square Maximum height of tabular roots (MHTR),  Number of tabular roots (NRT),  Maximum length of tabular roots (MLRT),  Perimeter at breast height (PBH),  Tree bark (TB).

Multiple correspondence analysis provided statistically significant support for 75.3% of the variation, revealing the presence of eight phlebotomine sand flies species *(Lu. serrana, Lu. gorbitzi, Lu. shannoni, Lu. evansi, Lu. ovallesi, Lu. c. cayennensis, Lu. rangeliana* and *Lu. atroclavata)* associated with trees with a circumference at breast height of 2.83 – 5.02 m, lacking of tabular roots and having laminar or fissured bark (Figure 3), all characteristics typical of the species recorded in the urban area of Sincelejo. Most immatures of *Lu. migonei* were collected from tree species with tabular roots between 2.5 m - 4.5 m tall, growing in the Coraza reserve. Immatures of *Lu. trinidadensis, Lu. pilosa, Lu. micropyga, B. hamata* and *B. mesai* found in tropical humid forest were associated with smooth-barked trees with 3–11 tabular roots that were 0.4 – 2.5 m tall and 1.1 – 2.6 m long (Figure 3).

### Physicochemical characterization of breeding site microhabitats

When physical parameters of the breeding sites were compared between the two ecosystems (Table 4), the median RH was found to be significantly higher in humid than in dry forests, at 93 vs. 89% (Mann–Whitney *U* test = 36; *P*-value = 0.017*). Temperatures of approximately 27°C were recorded in all microhabitats. Other variables, such as the capacity to retain water, suggest that the breeding sites are clay soil but with good filtration. These soils had a granular aspect characteristic of friable soils with a higher density in humid than in dry forest. The pH values recorded for humid and dry forest samples were approximately neutral to slightly alkaline and their conductivity values were propitious for the exchange of ions generated during the decomposition of organic material. With regard to the principal bioelements, nitrogen was more conserved in dry than in humid forest microhabitats and organic carbon levels were low in both ecosystems, while the median C/N ratio of the natural breeding sites was much higher than in humid forest.

**Table 4:** **Characterization of microhabitat variables and physico-chemical characteristics of soil samples from natural breeding sites of phlebotomine sand flies**

**Ecosystem, locality**	**Tropical humid forest**			**Tropical dry forest**			**Contrast test**	
	**Río Claro-El Aguacate**			**Sincelejo-Coraza**				
**Physicochemical variable**		**Percentile**			**Percentile**			
	**Median**	**25**	**75**	**Median**	**25**	**75**	**U-MWhitney**	**p value**
1. Relative humidity (%)	93	90	95	89	79	93	36	0.017*
2. Temperature (°C)	27.4	27.0	28.7	27.6	26.7	28.6	82.5	0.6
3. Nitrogen (%)	0.5	0.3	0.7	0.6	0.4	0.8	73	0.4
4. Organic carbon (%)	9.5	2.3	21.7	12.9	5.8	27.3	70	0.3
5. Carbon/Nitrogen	12.6	6.3	34.4	20.3	12.6	35.1	69	0.3
6. pH	7.4	7.3	7.7	7.5	6.8	7.9	92	0.9
7. Water-holding capacity (%)	98.9	56.2	184.8	114	77	150.8	77	0.5
8. Conductivity (uS/cm)	761	289.1	1106.5	10.2	2.5	1113	62	0.2
9. Density (g/cm3)	0.7	0.3	1.0	0.5	0.3	0.7	72.5	0.4

**Us/cm:** Microsiemens per centimeter; **g/cm** ^**3**^ **:** Grams per cubic centimeter.

* Significantly statistical value.

**Figure 4:**
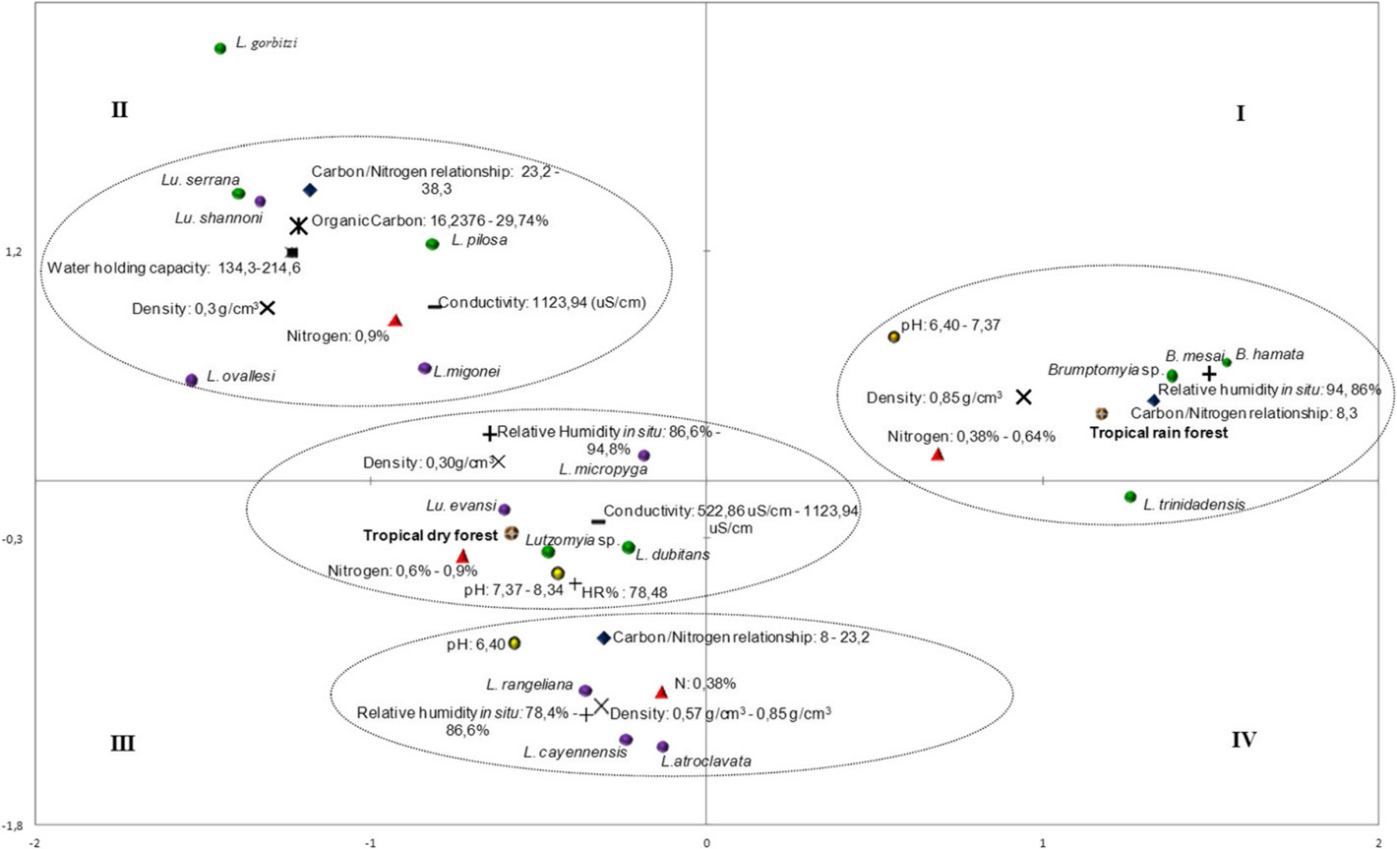
**Multiple correspondence analysis (foreground factorial axes F1 and F2, 59.74% of variance), which relates physicochemical variables of natural breeding places of phlebotomine sand flies in tropical dry forest ecosystems and humid tropical in Colombia.**  Ecosystem, green circle Species of the subfamily Phlebotominae that are not vectors of *Leishmania*, purple circle Species of the subfamily Phlebotominae associated with the transmission of *Leishmania*,  Organic Carbon,  Relative Humidity,  Conductivity, red triangle Nitrogen, black square Water holding capacity,  Density, dark blue diamond Carbon/Nitrogen,  pH.

The correlation of the phlebotomines with the physicochemical characteristics of different microhabitats, using multiple correspondence analysis (59.7% of the variation), indicates that the immatures of *Lu. trinidadensis, B. hamata* and *B. mesai* develop in breeding sites with high RH (94.9%) and neutral pH (6.4-7.4), containing substrates of high density (0.85 g/cm^3^), low C/N ratio (8.03) and high percentage nitrogen (0.38 - 0.64) (Figure 4). Species such as *Lu. atroclavata, Lu. rangeliana* and *Lu. c. cayennensis* develop in breeding sites that have similar values for pH, density and percentage nitrogen, but lower RH (74.5 - 86.7%) and higher C/N (32.1) (Figure 4). The physicochemical determinants for the presence of immatures of *Lu. evansi, Lu. micropyga* and *Lu. dubitans* differed from the two previous groups in terms of a high percentage of nitrogen (0.6-0.9), moderate conductivity (522.86 –1123.94 uS/cm) and more alkaline soil pH (7.4–8.3) (Figure 3). Finally, we suggest the presence of immatures of *Lu. serrana, Lu. shannoni, Lu. pilosa, Lu. ovallesi* and *Lu. migonei* in microhabitats with a high C/N ratio (38.4), percentage of organic carbon (29.7%) and water retention capacity (134.3–214.6).

## Discussion

Identification of natural breeding sites is an important topic in the biology of the phlebotomine sand flies. However, this requires collecting numerous substrate samples (soil, leaf litter etc.), which may contain larvae or pupae of these insects. For this reason, sampling and observation efforts must be exhaustive [12,13,21,25,26].

In this study, phlebotomine breeding sites are recorded for the first time in urban habitats, in the Colombian City of Sincelejo. These habitats are undergoing transformation with dwindling remnants of the original forest giving way to parks planted with large, non-native species, nurseries and abandoned lots with abundant vegetation, constituting intervened ecosystems to which sand flies may adapt [40,41]. It is noteworthy that in the urban habitat studied here, the bases of trees were significant as breeding sites, by finding mainly immatures of *Lutzomyia.*

This fact is important in the ecoepidemiology of Leishmaniasis because it involves equilibrium between zooprophylaxis, maintenance of sand fly populations and attraction of reservoir hosts. Spatial analysis studies are necessary to detect areas at increased risk for leishmaniasis, such as those analyses that include immature localization and productivity of breeding sites.

This contrasts with rural habitats such as the Río Claro, El Aguacate and Coraza reserves, where tabular roots and tree holes were the most favorable places for the development of immature phlebotomines (genus *Brumptomyia*). Similar findings were obtained in previous studies in Brazil and Panama [19,21]. The microhabitat characteristics that are suitable for development of immatures of genus *Lutzomyia* and *Brumptomyia* seem to include an abundance of organic material derived from animal faeces, decomposing leaves and fruit, as well as structures such as trees with protected spaces that provide refuge from rain, wind, strong light and predators [19-21,42].

It should be noted that in Antioquia (Reserve Rio Claro) and Chocó (Reserve El Aguacate), the number and types of potential breeding sites was lower when compared to the sampling conducted in the Colombian Caribbean coast because the sampling intensity was lower in terms of time and repetition. The forest environments of the two nature reserves (Tropical Humid Forest) had related endemic vegetation different to rural and urban areas of the Caribbean coast, indicate that the roots, tabular roots of trees, where the most concentrated adult collections were found by active search in diurnal resting sites. In addition, we did not search for immatures in tree bark because most trees are smooth and without striations, and this feature does not allow the accumulation of organic matter that may be useful for the development of immature *Lutzomyia*. In contrast to the urban area of Sincelejo, the tree composition where sampling efforts were concentrated consists of ornamental and introduced species with no tabular roots, but with laminar and fissured bark covered by vegetation favoring a microenvironment aggregation attractive to adults and suitable for the development of immature stages of *Lutzomyia*.

Microhabitats previously identified as phlebotomine breeding sites also include termite mounds and animal burrows, as documented by other studies in areas as diverse as arid areas of Kenya and the Brazilian Amazon [23,43,44]. Microhabitats with relatively stable temperatures and RH values can be found in all of these places, providing shelter and breeding sites for sand flies where they are protected from environmental factors such as light, wind and rain. On the other hand, it is also known that some immatures are more exposed to the environment, such as in leaf litter, and thus directly suffer the effects of luminosity, precipitation and contact with other species of invertebrates and vertebrates [21,28,45-50].

With respect to the association between phlebotomines and particular tree species, it should be taken into account that mature trees have features such as large trunks, fissured bark, and roots that project from the soil, which in turn may influence the degree of protection and the accumulation of organic material that determine the presence of immature phlebotomines [22,51]. The tree families associated with phlebotomine breeding sites include the Anacardiaceae, including *Anacardium excelsum*, a species that produces large quantities of leaf litter, which provides an important source of organic material and protection for the uppermost layers of the soil in which immature sand flies develop [22,52]. Another important family is the Bombacaceae [53], which includes species such as *Ceiba pentadra*, present in three of the four study areas, whose large tabular roots provided the greatest number of immatures collected during the present study, mainly of *Brumptomyia*.

The family Fabaceae includes several species recorded during the present study, i.e., *Pithecellobium dulce* Benth 1844, *Platymiscium pinnatum* Dugand 1938*, Samanea saman* Merrill 1916, *Pseudosamanea guachapele* Kunth, *Myroxylon balsamum* Harms 1887, and *Brownea ariza* Benth 1845, for sand flies. These trees have deep roots which bear secondary root nodules containing bacteria of the genus *Rhizobium*. These bacteria are able to fix sufficient nitrogen that would be available to soil biota - in such microhabitat phlebotomine larvae would take advantage of a rich soil to find nutritious food [54,55]. The fact that the sand flies require a wide variety of diets is due to nutritional deficiencies and the difficult digestion by the presence of complex molecules present in the soil in the case of larvae. In this context and since a metabolic point of view, communities of bacteria in the digestive tract are necessary for detoxification of plant material and promote the availability of Fe ++ erythrocytes, essential amino acids and vitamins B. Furthermore, these trees are large and have a symmetrical canopy [54,56,57], providing shade and ensuring stable temperature and RH values for breeding site microclimates.

The composition of tree species present in the two ecosystems differed significantly in certain morphological characteristics (number of tabular roots, type of bark), which may influence the breeding preferences of *Lutzomyia* and *Brumptomyia* sand flies. Although Ready et al. declared that tree trunk circumference is not a determinant of phlebotomine abundance [22,58], Geoffroy et al. noted that this was related to resting surface area and increased breeding site availability [59]. This is consistent with our finding that the greatest number of immature sand flies was associated with trees that had girths of 2–5 m.

The number, height and length of tabular roots also influence the development of the Phlebotomines of the genus *Brumptomyia*, offering stability and protection in dry periods when many deciduous trees shed their leaves, producing a seasonal reduction of the canopy [60]. In the urban zone of Sincelejo, where the trees lack tabular roots, grasses and other herbaceous plants might play a role in protecting the immature insects that develop in the bases and barks of the trees.

Although tree species and other environmental factors may influence the availability of phlebotomine breeding sites, physicochemical parameters evaluated in this study, such as pH, organic carbon content, nitrogen, C/N ratio, RH and temperature, are essential to larval survival and development. Physical properties of microhabitats, such as conductivity, soil density and water retention capacity, may also be important [24]. In general, the physicochemical characteristics of the two types of ecosystems in the present study were similar. RH measurements indicate that sand fly larvae prefer to live in soils that are humid but not inundated because they cannot survive or remain underwater for prolonged periods [25,61]. With respect to temperature, the stable values recorded in the present study are similar to those found by Rutledge and Ellenwood, who encountered temperature ranges of 22-27°C within the surface layers and interiors of 32 natural breeding sites in Panamanian forest [46,51]. According to Theodor, temperatures below 10°C and above 40°C are unfavorable to phlebotomine larvae [61].

Among the physical variables evaluated, the capacity to retain water suggests that sand fly breeding sites have the good drainage characteristics associated with loam soils, i.e., those with a homogenous sand/mud/clay ratio [25,62]. This characteristic is a determinant of larval survival because soils that are too sandy retain little water and dry out rapidly, while clay soils tend to become compacted and drain poorly, causing waterlogging and rapid death of the larvae [23,49,63,64]. In dry tropical forest ecosystems the soils of breeding sites are less dense and thus more porous, facilitating the circulation of air. Humid forest breeding sites have denser soils, with a less porous matrix that tends to accumulate more water due to poor drainage, which may explain the high RH values observed [62,63].

Dissolved nitrogen was available in the soil samples, indicating that breeding sites were systems that were undergoing continuous renovation. The availability of nitrogen resulting from decomposing plant material in field samples is essential for the nutrition and metabolism of phlebotomine larvae because this element is the basis for the synthesis of organic compounds (i.e., proteins, amino sugars) [24,62-64].

The high C/N ratio in dry tropical forest suggests that the breeding sites in the urban area of Sincelejo and the Coraza reserve have a thin upper phase of soil with a high carbon concentration. In this type of substrate, decomposition of organic material is slow and carried out by small arthropods such as millipedes and termites, as well as mesophilic microorganisms and fungi [19,62,63]. The C/N ratio in humid tropical forest is low, with a thicker organic material phase that has higher contents of available nitrogen, due to rapid transformation by microorganisms such as bacteria [62].

Finally, pH is the chemical variable that has been most studied for sand fly breeding sites to date [13,23,24]. The pH of the breeding sites examined in the present study was neutral to slightly alkaline in both ecosystems, this being characteristic of a stable niche that favours homeostasis, digestion of nutrients and ionic exchange by phlebotomine larvae [24]. Furthermore, pH may influence larval development by conditioning growth of soil micro-organisms as possible larval food.

The pH and physical structure of natural breeding sites are points that can be used to control immature of sand flies, but depends on the area of study in particular. For example, *P. orientalis* in Sudan and Ethiopia is associated with cracked black-cotton soil, which can be amended by adding gypsum or gypsum with Ca-zeolites [15,64,65]. Although some studies suggest adding lime to the natural breeding sites, this strategy may be more complicated and controversial, by large amounts of lime to raise the pH considerably and the ecological impact it can generate in other biological communities.

Control of larvae in soil habitats may also be achieved with organic insecticidal (soil fertilizers), such as Neem oil-cakes (contains Azadirachtin) potentially useful to kill sand fly larvae [15,66]. Alternatively, *Argemone mexicana L.* has been shown to produce organic acids on decomposition and can be used to treat alkaline soils (reduce soil pH) [13], which might help in preventing vector breeding, and be a useful alternative to chemical insecticides for sand fly control.

## Conclusions

The intrinsic characteristics of breeding sites could help identify ecological markers for phlebotomine development. However, studies on intrinsic characteristics of breeding sites in the peridomicile that have focused on the physical change of the habitat or the use of chemical control of sand flies on larval and pupal stages are worthy of being evaluated. In this context, our results show a wide range of ecological variables associated with breeding sites of immature stages, which may potentially be of use for the design of future vector control measures for *Leishmania* transmission in environments of tropical wet and dry forests in Colombia.

## Abbreviations

*Lu*: *Lutzomyia*
*Br*: *Brumptomyia*
HTF: Humid tropical forest
TDF: Tropical dry forest
NBE: Number of natural breeding examined
INM: Number of immature
NB+: Positive natural breeding site with presence of immatures
Cm: Centimeter
L: Liter
g: Grams
kg: Kilogram
RH: Relative humidity
mL: Milliliter
COI: Cytochrome oxidase I
C/N: Carbon nitrogen ratio
